# Rapid cardiac thermal acclimation in wild anadromous Arctic char (*Salvelinus alpinus*)

**DOI:** 10.1242/jeb.244055

**Published:** 2022-09-09

**Authors:** Matthew J. H. Gilbert, Ella K. Middleton, Kevin Kanayok, Les N. Harris, Jean-Sébastien Moore, Anthony P. Farrell, Ben Speers-Roesch

**Affiliations:** ^1^ Department of Zoology, University of British Columbia, #4200-6270 University Blvd, Vancouver, BC, Canada, V6T 1Z4; ^2^ Department of Biological Sciences, University of New Brunswick – Saint John, 100 Tucker Park Road, Saint John, NB, Canada, E2L 4L5; ^3^ Ekaluktutiak Hunters & Trappers Organization, Box 1270, Ekaluktutiak, NU, Canada, X0B 0C0; ^4^ Arctic and Aquatic Research Division, Fisheries and Oceans Canada, 501 University Crescent, Winnipeg, MB, Canada, R3T 2N6; ^5^ Institut de Biologie Intégrative et des Systèmes and Département de Biologie, Université Laval, 1030 Avenue de la Médecine, Quebec City, QC, Canada, G1V 0A6; ^6^ Faculty of Land and Food Systems, University of British Columbia, 2357 Main Mall, Vancouver, BC, Canada, V6T 1Z4

**Keywords:** Heart rate, Thermal variation, Acclimation rate, Climate change, Thermal tolerance, Cardiac function

## Abstract

Migratory fishes commonly encounter large and rapid thermal variation, which has the potential to disrupt essential physiological functions. Thus, we acclimated wild, migratory Arctic char to 13°C (∼7°C above a summer average) for an ecologically relevant period (3 days) and measured maximum heart rate (ƒ_H,max_) during acute warming to determine their ability to rapidly improve cardiac function at high temperatures. Arctic char exhibited rapid compensatory cardiac plasticity similar to past observations following prolonged warm acclimation: they reduced ƒ_H,max_ over intermediate temperatures (−8%), improved their ability to increase ƒ_H,max_ during warming (+10%), and increased (+1.3°C) the temperature at the onset of an arrhythmic heartbeat, a sign of cardiac failure. This rapid cardiac plasticity may help migrating fishes such as Arctic char mitigate short-term thermal challenges. Furthermore, by using mobile Arctic research infrastructure in a remote field location, the present study illustrates the potential for field-based, experimental physiology in such locations.

## Teaser

**Summary:** Wild migrating Arctic char can rapidly adjust the temperature sensitivity of their hearts; this rapid cardiac thermal plasticity may help migratory fishes like Arctic char mitigate short-term thermal challenges.

## INTRODUCTION

Many fishes, including many Arctic species, can experience rapid and large spatiotemporal thermal variation. Such rapid variation occurs with movement through thermally heterogeneous environments (e.g. −2 to 10°C over minutes to hours; Harris et al., 2020b), with diurnal or weather-related fluctuations (e.g. >10°C fluctuation within a day; Gilbert et al., 2016; Gilbert and Tierney, 2018). Anadromous Arctic char (*Salvelinus alpinus*) are a remarkable example in this regard because, after spending the summer in the Arctic Ocean at ∼6°C, their upriver migration to their spawning and overwintering areas requires passage through rivers with temperatures that range from 0 to 21°C (Gilbert et al., 2016; Harris et al., 2020a,b). Such abrupt thermal variation is likely to challenge the physiological functions and thus whole-organism performance of any migratory fish including Arctic char.

Heart rate (ƒ_H_) is one critical function that is strongly influenced by temperature in fishes (Farrell and Smith, 2017). Warming typically drives an exponential increase in routine ƒ_H_ (ƒ_H,routine_) to support an exponential rise in routine oxygen consumption (Eliason and Anttila, 2017; Farrell, 2016). Correspondingly, maximum heart rate (ƒ_H,max_) must also increase with temperature to maintain the ability to elevate ƒ_H_ above ƒ_H,routine_ (i.e. scope for heart rate) in support of functions such as digestion and exercise. However, acute warming only increases ƒ_H,max_ until a peak is reached, beyond which, ƒ_H,max_ typically declines and the heartbeat ultimately loses its rhythmicity (Casselman et al., 2012; Eliason and Anttila, 2017; Farrell, 2009; Vornanen, 2016). Prolonged warm acclimation (weeks) for many fish species, including Arctic char, can help compensate by reducing ƒ_H_ (ƒ_H,routine_ and ƒ_H,max_) over moderate temperatures and improving its stability at high temperatures (Aho and Vornanen, 2001; Badr et al., 2016; Eliason and Anttila, 2017; Gilbert and Farrell, 2021; Vornanen, 2016). Yet, the extent to which such cardiac thermal plasticity occurs more rapidly (Ekström et al., 2016) and in phase with natural acute thermal variation remains much less clear. Recently, juvenile lab-reared rainbow trout (*Oncorhynchus mykiss*) were shown to improve their ability to increase ƒ_H,max_ during acute warming with a warm acclimation of just 24 h, and by 72 h they exhibited a significant thermal compensation (i.e. reduction) of ƒ_H,max_ (Gilbert et al., 2022). Likewise, Sutcliffe et al. (2020) observed a resetting of intrinsic ƒ_H_ after only 1 h of warm acclimation in lab-reared rainbow trout. Thus, in the absence of field studies, it seems plausible that rapid cardiac plasticity in wild fishes such as anadromous Arctic char could help mitigate natural acute thermal warming challenges experienced during river migrations.

Consequently, we examined whether wild sea-run adult Arctic char could rapidly adjust the thermal performance of their heart during a brief (∼72 h) acclimation to 13°C, which is ∼7°C above their typical summer marine average. We hypothesized that Arctic char could rapidly acclimate cardiac function to a warmer temperature. If our hypothesis was correct, we expected three specific results: (1) Arctic char would lower ƒ_H,max_ over cool and intermediate temperatures, counteracting the positive chronotropic effect of warming, (2) they would improve their ability to increase or maintain ƒ_H,max_ at high temperatures, and (3) they would increase the temperatures at which peak ƒ_H,max_ and cardiac arrhythmia first occurred. The logistical challenges of performing such physiological measurements on wild Arctic char in the central Canadian Arctic were solved by using innovative mobile research infrastructure. Thus, a secondary aim of our study was to demonstrate the utility of and need for mobile research infrastructure to address pressing research priorities in remote regions.

## MATERIALS AND METHODS

We established an ecologically relevant duration for warm acclimation by tracking the duration of the upriver migration for 19 Arctic char, *Salvelinus alpinus* (Linnaeus 1758), at Halokvik, NU, Canada (69.175°N 107.104°W) in August 2017 as part of a longer-term Arctic char tracking program in the region (Moore et al., 2016). At the start of their upriver migration, these fish were implanted with acoustic tags (V16, InnovaSea, Bedford, NS, Canada) as previously described (Moore et al., 2016). An acoustic receiver (VR2AR, InnovaSea) was deployed at Pangniktok (South Lake, NU, Canada; 69.272°N 108.065°W; Fig. S1), the first lake Arctic char encounter during their upriver migration, although some fish continue further from there at presumably cooler temperatures. Thus, the minimum individual migration duration was taken as the difference between the time of release and the time of the first detection at Pangniktok. Temperature was recorded (HOBO pendant logger; Onset, Bourne, MA, USA) in the lower 1 km of the river every 15 min for the duration of this migration period. Temperature data (recorded every 5 min) were also available from the same location for 2013 from related research (Gilbert, 2020; Harris et al., 2020a). The Halokvik upriver migration is one of the longest (>50 km) in the central Canadian Arctic and among the most thermally variable (Gilbert, 2020). Based on the observed migration durations and temperature profiles, we selected 3 days (68–75 h) and ∼13°C as a conservative acclimation duration and temperature.

The thermal acclimation experiments were conducted at Palik (Byron Bay at the mouth of Lauchlan River, NU, Canada; 68.945°N, 108.532°W; Fig. S1) during July 2019 (*n*=12) and August 2021 (*n*=12), using mobile research infrastructure (Arctic Research Foundation, Winnipeg, MB, Canada) which has been previously described (Gilbert et al., 2020). Palik is a remote field location >140 km by water from the nearest settlement and >1500 km from the nearest aquatics research facility, but just ∼70 km west of Halokvik by water, and Arctic char commonly move between the two locations while feeding in the ocean over summer (Moore et al., 2016, 2017). The present study is the first to use a mobile laboratory for a multi-day thermal acclimation study in the Arctic.

Wild anadromous adult Arctic char (3245±726 g, mean±s.d.) were caught through angling or continuously watched gill nets (139 mm). Arctic char were immediately transferred to submerged mesh holding pens (∼1 m^3^) and then some were transferred by cooler (∼100 l) to the temperature-controlled holding system (∼500 l) in the near-shore mobile laboratory. Up to four adult Arctic char were held in the holding system at a time; they were transferred to the system at their ambient water temperature (7–9°C) and were warmed at 1–2°C h^−1^ to the acclimation temperature of ∼13°C. Fish were held at ∼13°C (13.3±0.2°C) for ∼3 days (68–75 h; hereafter referred to as ‘warm acclimated’), during which time water temperature was continuously recorded. Dissolved oxygen was maintained at >70% air saturation and large (∼50%) water exchanges were performed, and Prime water conditioner was added (as per the manufacturer’s instructions, Seachem, Madison, GA, USA) 1–2 times daily depending on ammonia levels. Most warm-acclimated fish performed well in the thermal performance assessments and were vigorous during handling immediately prior to testing. Two mortalities occurred during holding at 13°C in both years, possibly from a combination of confinement stress and water quality-related issues. Thus, future holding studies in remote, space-limited systems such as ours might benefit from the use of smaller sized fish at the same life stage, more frequent water exchanges and additional filtration.

We examined the extent of cardiac plasticity following the brief warm acclimation, by measuring the response of ƒ_H,max_ to acute warming as previously described (Gilbert et al., 2020) in non-acclimated (i.e. control fish; *n*=15) and warm acclimated (*n*=9) Arctic char. Briefly, we anaesthetized (150 mg l^−1^ tricaine methane sulfonate, TMS, buffered with 225 mg l^−1^ NaHCO_3_) individual fish at ambient water temperature (∼7–9°C) and then transferred them to the experimental bath containing a well-aerated maintenance anaesthetic solution (75 mg l^−1^ TMS buffered with 112.5 mg l^−1^ NaHCO_3_) at 5°C. We continuously irrigated their gills with the bath solution, fitted them with subdermal electrodes and delivered intraperitoneal injections of atropine (1.2 mg kg^−1^) and isoproterenol (4 μg kg^−1^) saline solutions (0.8% NaCl; total volume of 1 ml kg^−1^) to elicit their ƒ_H,max_ (Anttila et al., 2014; Casselman et al., 2012; Gilbert et al., 2020). We recorded and analysed an electrocardiogram (ECG as previously described; Gilbert et al., 2020). The ƒ_H,max_ was allowed to stabilize for at least 20 min before the bath was warmed at 5–6°C h^−1^ in 1°C increments until the heartbeat became arrhythmic and the experiment was terminated. We made minor adjustments to electrode placement as needed throughout the protocol as ECG quality can change with warming. The ƒ_H,max_ was recorded over the final minute of each 1°C warming increment. In all cases, ƒ_H,max_ increased with acute warming, reached a peak, and then began to decline prior to the onset of arrhythmia. Cardiac arrhythmias at high temperatures were apparent as entirely missing or delayed QRS complexes (ventricular depolarizations) within the ECG trace, and as such resulted in a further and more dramatic collapse of ƒ_H,max_ (Fig. S2). Among all observed arrhythmias, 35% were classified as a severe atrioventricular (AV) block type arrhythmia, defined as one or more P-waves (indicating atrial depolarization) with missing QRS complexes (indicating an absence of ventricular depolarization; Fig. S2A,B). The rest were characterized by either completely missing or severely delayed heartbeats (no P-wave or QRS-wave; Fig. S2C,D). These large gaps in the ECG traces (Fig. S2C,D) generally at least halved the instantaneous *f*_H,max_ (i.e. doubling of the R–R interval).

**Fig. 1.**
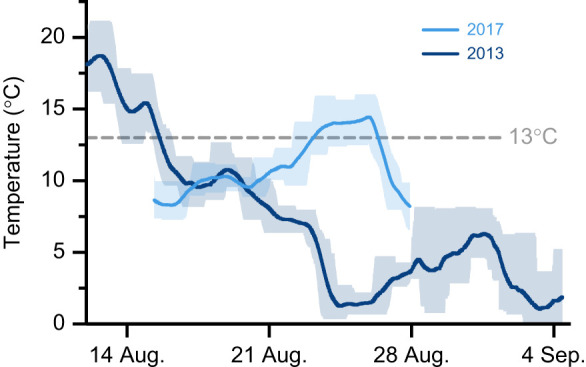
**River temperatures at Halokvik, NU, Canada, during research on the upriver Arctic char migration in 2013 and 2017.** Data are presented as the daily mean (solid line), minimum and maximum (upper and lower shading).

An incremental *Q*_10_ temperature coefficient for ƒ_H,max_ was calculated over each 2°C increase (Anttila et al., 2013, 2014) because the relative thermal sensitivity of ƒ_H,max_ commonly decreases with increasing temperature (Fig. 1). This decline in *Q*_10_ with warming is contrary to the simple exponential rise implied when calculating a single *Q*_10_ value over a 10°C range of temperature. A declining incremental *Q*_10_ can be interpretated as a declining ability to increase ƒ_H,max_ with further acute warming (Anttila et al., 2013).

We used three indicators of cardiac thermal performance: the temperature when the incremental *Q*_10_ fell below 1.5 (*T_Q_*_10<1.5_), the temperature at peak ƒ_H,max_ (*T*_peak_) and the temperature when a cardiac arrhythmia first appeared (*T*_arr_). The peak ƒ_H,max_ attained during warming and the total change in ƒ_H,max_ (Δƒ_H,max_=peak ƒ_H,max_−ƒ_H,max_ at 5°C) were also assessed as indicators of the peak and total ability to increase ƒ_H,max_ to support acute warming. Two warm-acclimated fish were excluded from the data analysis because post-experiment dissection of one revealed a severe cardiac parasitic infection, and the other was lethargic during acclimation and its heart rate was unstable prior to the start of the test. Fish capture and procedures were approved by the Fisheries and Oceans Canada Freshwater Institute (FWI-ACC AUP-2021-48 and 2019-37).

Data presentation was completed with Prism v.9 (GraphPad Software, San Diego, CA, USA) and data analysis with R Studio (α=0.05; http://www.R-project.org/). Changes in ƒ_H,max_ and the incremental *Q*_10_ during acute warming were characterized using linear mixed effects models (lme4 package; https://CRAN.R-project.org/package=lme4) with each modelled as a function of acclimation status, acute temperature, and fish ID as a random effect (Table S1). The fixed-factor interaction was excluded in both cases because there was limited or no evidence (*P*>0.05) that it improved the model fit (i.e. lowered AIC). The increase in ƒ_H,max_ was modelled from 5 to 17°C as ƒ_H,max_ stopped increasing above 17°C in some individuals. Body mass was log-transformed and included as a covariate to account for any potential negative allometric scaling of ƒ_H_ (Clark and Farrell, 2011). Coefficients of determination (*R*^2^) were calculated for each model with (conditional *R*^2^) and without (marginal *R*^2^) the random effect included using the MuMIn: Multi-Model Inference package (https://CRAN.R-project.org/package=MuMIn). Differences in response variables (ƒ_H,max_ at 5°C, peak ƒ_H,max_, Δƒ_H,max_, *T_Q_*_10<1.5_, *T*_peak_ and *T*_arr_) between the two acclimation treatments were assessed with an analysis of covariance which included log_10_(body mass) as a covariate. If there was limited evidence (*P*>0.05) for an effect of body mass, Student's *t*-test was performed to assess the treatment effect with a one-tail test specific to our directional, *a priori* predictions for each metric (see Introduction). Assumptions of normality and homogeneity of variance were verified using Shapiro–Wilk and Levene tests, respectively. We used a Fisher's exact test to assess whether there was a difference in the prevalence of severe AV block type arrhythmias (Fig. S2) between the treatment and control groups. Data are presented as means±s.e.m. unless otherwise noted.

## RESULTS AND DISCUSSION

### Migration duration and river temperatures

Of the 19 Arctic char implanted with acoustic tags, 14 were detected upriver after completing their migration. Their migration duration was 7.1±2.9 days (mean±s.d.; range: 3–13 days). In 2017, the maximum, minimum and mean (±s.d.) temperatures during the migration period in the lower reaches of the river at Halokvik were 16.0, 6.6 and 10.8±2.3°C, with a maximum 3 day average of 14.1±1.1°C (Fig. 1). In August 2013, during related research (Harris et al., 2020a), the maximum, minimum and mean (±s.d.) temperatures were 21.2, −0.8 and 7.3±5.3°C, with a maximum 3 day mean of 17.1±2.3°C (Fig. 1).

### The response of *f*_H,max_ to acute warming

**Fig. 2.**
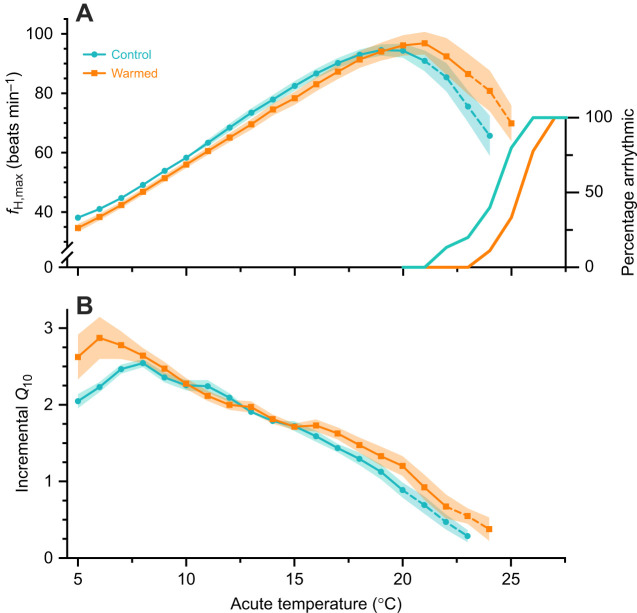
**The response of maximum heart rate (ƒ_H,max_) to acute warming in wild adult Arctic char with and without (control) a 3** **day warm acclimation (∼13°C).** The mean (±s.e.m., shading) ƒ_H,max_ (A) and corresponding incremental *Q*_10_ (B) are shown during acute warming in control (*n*=15) and warmed (*n*=9) fish, with dashed lines indicating temperatures at which individuals were excluded after exhibiting cardiac arrhythmias. Model statistics are presented in Table S1. The inset in A shows the progressive increase in the percentage of individual fish showing a cardiac arrhythmia at a supra-optimal temperature.

**Fig. 3.**
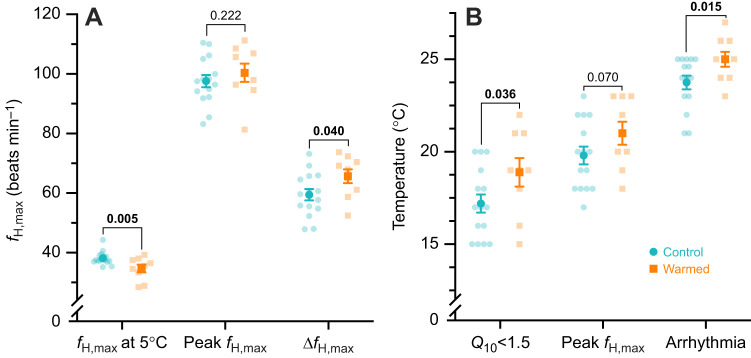
**The effect of a 3 day warm acclimation on ƒ_H,max_ and transitional temperatures for ƒ_H,max_ during acute warming.** Mean (±s.e.m.) and individual values (semi-transparent) are shown for (A) ƒ_H,max_ at 5°C, the peak ƒ_H,max_ and the difference between the two (Δƒ_H,max_), and (B) the temperatures at which incremental *Q*_10_ fell below 1.5 (*T_Q_*_10<1.5_), peak ƒ_H,max_ occurred (*T*_peak_) and the heart became arrhythmic (*T*_arr_). *P*-values for the effect of warm acclimation (*t*-test) are presented above each metric with significant differences in bold.

The ƒ_H,max_ increased with acute warming in all individuals (Fig. 2A). As predicted, ƒ_H,max_ was reset to a lower rate in warm-acclimated fish (an overall decrease of 8%; Fig. 2A; Table S1; *t*_22_=−2.459, *P*=0.023) over the intermediate temperatures (from 5 to 17°C). For example, at the start of acute warming, ƒ_H,max_ at 5°C was 9% lower with warm acclimation compared with controls (34.7±1.3 versus 38.2±0.6 beats min^−1^, *t*_22_=2.80, *P*=0.005; Fig. 3A).

Cardiac heat tolerance and the ability to increase ƒ_H,max_ during acute warming were also improved following warm acclimation. As expected, the incremental *Q*_10_ declined during acute warming in all individuals (Fig. 2B; Table S1). However, the decrease with temperature was less pronounced with warm acclimation (Table S1; *t*_22_=3.343, *P*=0.003). Consequently, warm-acclimated fish had a greater overall *Q*_10_ from 5 to 15°C (2.27±0.04 versus 2.16±0.03, *t*_22_=−2.01, *P*=0.029) and *T_Q_*__10_<1.5_ (+1.7°C, *F*_1,22_=5.027, *P*=0.036; Fig. 3B). Peak ƒ_H,max_ did not significantly increase (+3%, *t*_21_=−1.036, *P*=0.156) after warm acclimation and we found only weak evidence of an increase in *T*_peak_ (+1.2°C, *t*_22_=−1.527, *P*=0.071; Fig. 3). However, as expected the total Δƒ_H,max_ achieved during acute warming and the *T*_arr_ were significantly greater (Δƒ_H,max_: +10% *F*_1,22_=4.780, *P*=0.040; *T*_arr_: +1.3°C, *t*_22_=−2.18, *P*=0.015; Fig. 3). Notably, we found weak evidence that AV block may be less prevalent (Fisher's exact test; *P*=0.086) in warm-acclimated Arctic char (1 of 8; one fish was excluded because of an ambiguous P-wave prior to the onset of arrhythmia) relative to control Arctic char (8 of 15).

### Summary of findings

Over the summer alone, anadromous Arctic char in the Kitikmeot region of Nunavut can experience a drastic range of temperatures from −1 to >21°C (Fig. 1 of the present study; Gilbert, 2020; Gilbert et al., 2016; Harris et al., 2020a,b). Furthermore, we demonstrated that peak temperatures encountered by Arctic char during their physically demanding upriver migration can reach 21°C with sustained temperatures (3 day average) as high as ∼17°C, while the migration can take >7 days. Thus, our acclimation duration (∼3 days) and temperatures (∼13°C) are not only ecologically relevant but also a conservative rather than extreme scenario, and rapid thermal plasticity could help mitigate such thermal challenges. Indeed, after just 3 days of warm acclimation, wild, migrating Arctic char in the present study exhibited significant thermal compensation of ƒ_H,max_ cardiac thermal sensitivity, and an improvement in cardiac heat tolerance.

During prolonged (weeks) warm exposure, many fishes, including Arctic char and other salmonids, decrease ƒ_H,max_ at a given temperature to counteract the effect of warming. For instance, ƒ_H,max_ was 12% lower at 12°C in Atlantic salmon (*Salmo salar*) acclimated to 20°C rather than 12°C for 3 months (Anttila et al., 2014). Likewise, ƒ_H,max_ when measured at 6°C was 19% lower in hatchery-reared Arctic char acclimated for >6 weeks to 14°C compared with those acclimated to 6°C (Gilbert and Farrell, 2021). Thus, our observation of an ∼8% reduction in ƒ_H,max_ over intermediate temperatures (5–17°C) following just a 3 day warm acclimation period suggests that this cardiac thermal compensation begins rather quickly when wild Arctic char encounter warm temperatures. Congruently, Gilbert et al. (2022) recently showed that roughly half of the total thermal compensation of ƒ_H,max_ (∼8%) in laboratory-reared rainbow trout acutely transferred from 10 to 18°C occurred within the first 3 days; further compensation then occurred more gradually over the following 25 days (∼17% total over 28 days). In other fishes, the thermal compensation of the pacemaker rate during warm acclimation is thought, in large part, to be achieved through a reduction in the delayed rectifier K^+^ current (*I*_Kr_), which reduces ƒ_H_ by lengthening the pacemaker action potential duration (Haverinen and Vornanen, 2007; Vornanen, 2016). A reduction in *I*_Kr_ could also account for the resetting of ƒ_H,max_ seen here. Consequently, the time course for warm acclimation of *I*_Kr_ and its association with ƒ_H,max_ warrants further investigation.

Our findings for a field study with wild Arctic char are generally consistent with the rapid increase in peak ƒ_H,max_ and Δƒ_H,max_ seen in rainbow trout (+15% and +28%, respectively) within 24 h of transfer from 10 to 18°C (Gilbert et al., 2022). Arctic char, however, did not increase peak ƒ_H,max_ with warm acclimation, but the combined reduction of ƒ_H,max_ (i.e. compensation) and maintenance or slight improvement in peak ƒ_H,max_ meant that the total Δƒ_H,max_ was improved by 10%. This increase in Δƒ_H,max_ stems from maintaining acute thermal sensitivity (i.e. delaying cardiac failure) after warm acclimation, as indicated by the elevations in incremental *Q*_10_ and *T_Q_*__10_<1.5_. Indeed, much longer warm acclimation periods for lab-reared Arctic char, rainbow trout, chinook salmon (*O. tshawytscha*) and Atlantic salmon (Anttila et al., 2014; Gilbert et al., 2022; Gilbert and Farrell, 2021; Muñoz et al., 2015) have produced similar changes. Nevertheless, the 10% improvement in Δƒ_H,max_ after 3 days of warm acclimation found here is modest compared with the 45% change seen for hatchery-reared Arctic char after a longer acclimation period from 6°C to 14°C (+45%; Gilbert and Farrell, 2021). As such, wild migrating Arctic char may show further cardiac plasticity during warm acclimation if the migration exposure is longer.

Cardiac heat tolerance of Arctic char can also markedly improve with warm acclimation. With prolonged acclimation, hatchery-reared Arctic char (from an anadromous source population only ∼75 km from our study site) can progressively increase *T*_arr_ and *T*_peak_ with acclimation temperatures from 2 to 14°C (Gilbert and Farrell, 2021). Specifically, *T*_arr_ and *T*_peak_ increased by ∼4 and 6°C following a >6 week acclimation to 14°C when compared with a 6°C acclimation temperature. Given that *T*_arr_ increased by 1.3°C after just 3 days of warm exposure, such acclimatory changes can certainly occur rapidly but may require longer to reach their full extent. Such a rapid initiation of warm acclimation agrees with a previous study on sheepshead minnows (*Cyprinodon variegatus*), in which ∼50% of the total observed increase (∼6.3°C) in whole-animal thermal tolerance occurred within 48–72 h following a transfer from 11 to 18°C (Fangue et al., 2014).

Mechanisms of prolonged cardiac thermal acclimation have received significant attention (Keen et al., 2017; Klaiman et al., 2011; Vornanen, 2016), but further research is needed to resolve the extent to which they contribute to the rapid plasticity in cardiac heat tolerance seen here. Heat-induced bradycardia and subsequent severe arrhythmias can be, at least in part, attributed to a mismatch in excitability among cardiac tissues (i.e. a source–sink mismatch) at high temperatures (Vornanen, 2016, 2020). Changes in the underlying ion currents (namely Na^+^ and K^+^) are thus an important aspect of cardiac thermal acclimation but the time course for such changes is not well established (Sutcliffe et al., 2020). A change in β-adrenergic receptor sensitivity or density could also have been a factor. β-Adrenergic stimulation can facilitate cardiac heat tolerance, β-adrenergic control can change with warm acclimation (Aho and Vornanen, 2001; Eliason et al., 2011; Gilbert et al., 2019), and we pharmacologically activated β-adrenergic receptors in the present study. While these types of acclimatory processes were likely at play here, given the short time frame of our acclimation, short-term heat stress responses may have also contributed to improving heat tolerance. Indeed, both wild-migrating and lab-reared Arctic char induce a rapid and pronounced heat shock response (e.g. induction of heat shock proteins) (Gilbert, 2015; Quinn et al., 2011) at sub-lethal temperatures, which could help stabilize cardiac function at higher temperatures. By improving or stabilizing ventricular excitability at high temperatures, any of these mechanisms could also account for a reduction in the prevalence of AV block type arrhythmias (Vornanen, 2016), which we found weak evidence for in warm-acclimated Arctic char.

### Conclusions

We show for the first time that wild Arctic char, despite being considered a cold-water specialist, can rapidly adjust their cardiac thermal performance during warm acclimation, well within a time frame that could help them cope with thermal challenges during migration. While these changes were modest relative to those possible with prolonged warm acclimation, any improvement in performance at warm temperatures could improve the proportion of fish able to complete their essential upriver migration to spawn and overwinter. Such rapid plasticity could also be important for other fishes, such as Pacific salmon (*Oncorhynchus* spp.), which also encounter temperatures that constrain their cardiorespiratory performance during their upriver migrations to their spawning areas (Eliason et al., 2011; Farrell, 2016). Thus, when taken together with past research on thermal acclimation rates in fishes (Ekström et al., 2016; Fangue et al., 2014; Gilbert et al., 2022), we demonstrate the need to consider relevant time courses for natural thermal variation when investigating the conservation or ecological implications of thermal acclimation. Here, we considered a natural time course for acclimation but if cold-water refugia are available, migrating Arctic char, like other salmonids, may behaviourally thermoregulate (Ebersole et al., 2003; but see Barrett and Armstrong, 2022). Such regulation could blunt the stimulus for warm acclimation. Thus, further research should examine how cardiac thermal plasticity manifests in fish migrating through thermally heterogeneous environments, and how acclimation rates and associated mechanisms are influenced by temperature parameters, fish health, life-history stage, population and species. Our remote field study (>1500 km away from the nearest aquatics facilities without road access), would not have been possible without innovative mobile Arctic research infrastructure, illustrating that investing in the development and deployment of such infrastructure can greatly expand basic and applied research possibilities in the north.

## Supplementary Material

10.1242/jexbio.244055_sup1 Supplementary information
