# Supplementary material for: Rapid cardiac thermal acclimation in wild anadromous Arctic char (Salvelinus alpinus)
Source: J Exp Biol. 2022 Sep 9;225(17):jeb244055. doi: 10.1242/jeb.244055 (PMC10215809; doi:10.1242/jeb.244055)
Supplement: Supplementary information [file jexbio-225-244055-s1.pdf]

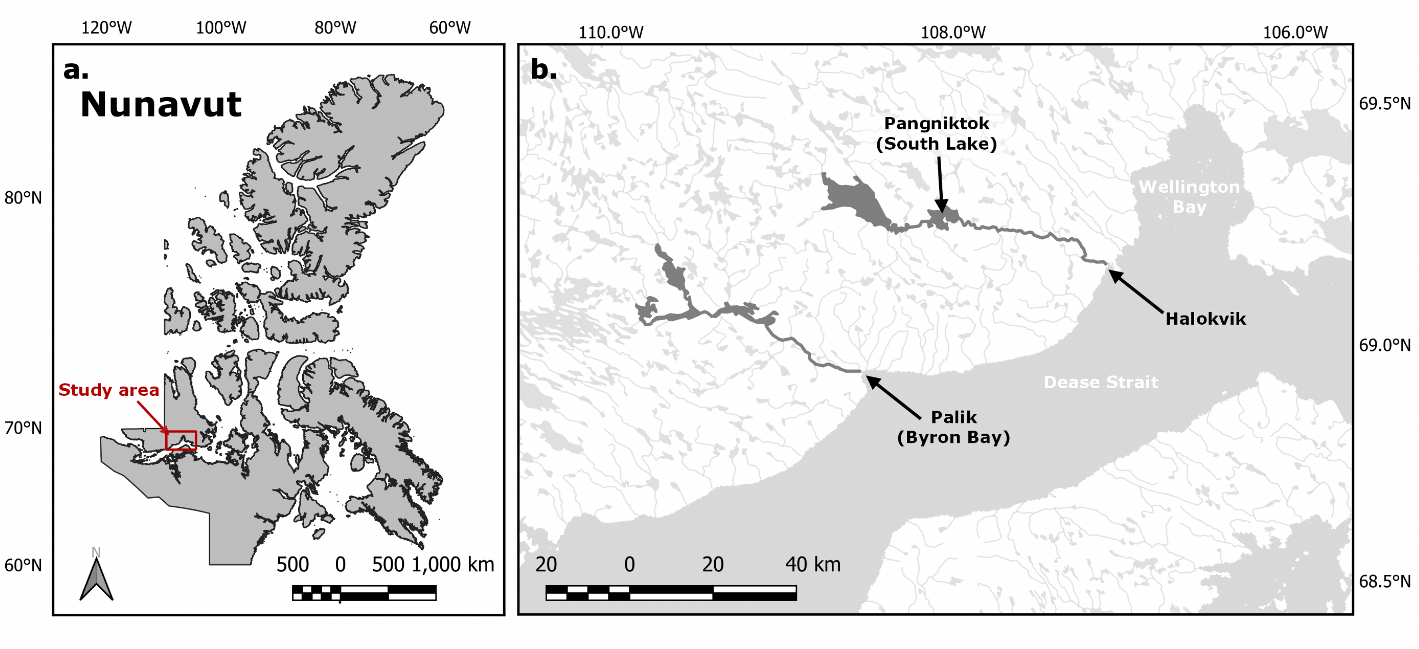

Fig. S1. The study area (red box in a, and enlarged in b) in the Kitikmeot Region of Nunavut, Canada. The acoustic telemetry work characterized the migration duration between Halokvik and Pangniktok (b). The cardiac thermal acclimation experiment was performed using a mobile laboratory at Palik.

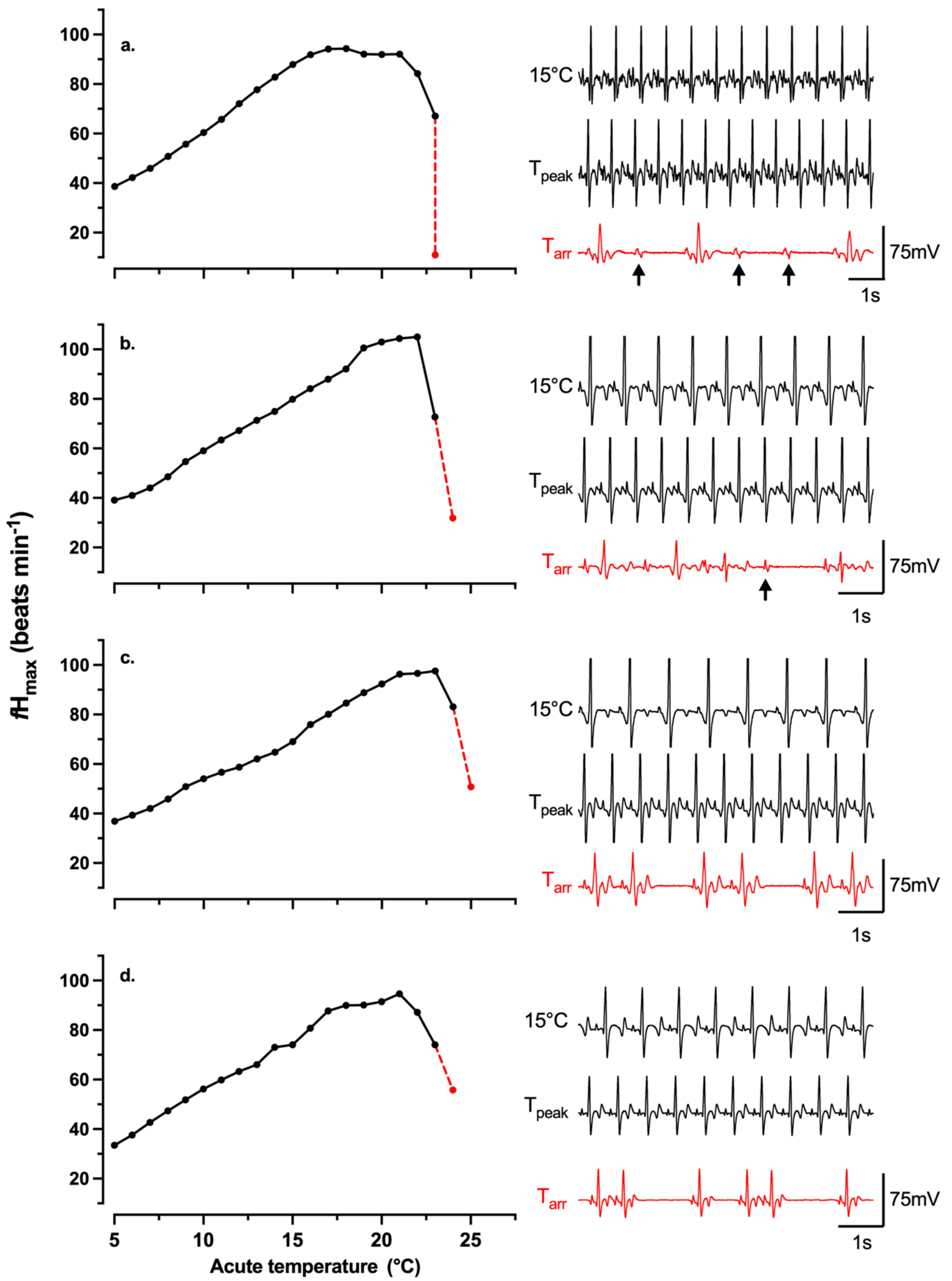

Fig. S2. The response of maximum heart rate (f_{Hmax}) to acute warming and representative heat induced changes in the electrocardiograms of four individual Arctic char (a-d). The f_{Hmax} during warming are shown (black points) with the f_{Hmax} at the onset of the cardiac arrhythmia indicated by a red point and the drop from the last rhythmic f_{Hmax} measurement with a dashed red line. Corresponding electrocardiogram traces are shown at 15°C and the temperatures at peak f_{Hmax} (T_{peak}) and arrhythmia (T_{arr}). Arrows mark unambiguous P-waves with no QRS complex, which indicates a severe AV-block (a and b).

Journal of Experimental Biology • Supplementary information

Table S1. Linear mixed effect model statistics for the change in maximum heart rate (fHmax) and incremental Q10 for fHmax during acute warming in control and warm acclimated (treatment) wild Arctic char. The fHmax was modelled from 5-17°C and Q10 from 5-25°C. Fish ID was included as a random effect.

	Coefficient	SE	Df	t	p
f_{Hmax}(beats min^{-1})					
Intercept	75.737	32.973	1, 21	2.297	0.032
Acute temp. (°C)	4.511	0.033	1, 287	135.077	<0.001
Warm acclimation	-4.908	1.996	1, 21	-2.459	0.023
Log_{10}(mass)	-17.421	9.310	1, 21	-1.871	0.075
			marginal R^2 = 0.93, conditional R^2 = 0.98		
Q_{10} for f_{Hmax}					
Intercept	3.482	0.050	1, 225	69.461	<0.001
Acute temp. (°C)	-0.127	0.003	1, 414	-42.067	<0.001
Warm acclimation	0.138	0.042	1, 22	3.343	0.003
			marginal R^2 = 0.80, conditional R^2 = 0.81		
